# Commentary: Why Are no Animal Communication Systems Simple Languages?

**DOI:** 10.3389/fpsyg.2021.763445

**Published:** 2021-10-15

**Authors:** Sławomir Wacewicz

**Affiliations:** Center for Language Evolution Studies, Nicolaus Copernicus University in Toruń, Toruń, Poland

**Keywords:** language evolution, animal communication, conflict of interests, Hockett's design features, platform of trust

## Introduction

In the title of his paper, Beecher (2021) asks “Why Are No Animal Communication Systems Simple Languages?” In his answer to this question, he identifies two necessary conditions for developing a language-like communication system: “strong cognitive and signal production mechanisms” and a low level of conflict of interests between the communicators. Although this answer is not qualitatively novel, Michael Beecher makes a highly valuable point in stressing this latter condition over the former one: while many animal species have a level of cognitive sophistication that should predispose them to have at least rudimentary languages, such species do not meet the other criterion, that of sufficient alignment of interest. I agree with the essence of this argument, which is still underappreciated in the language evolution literature. However, I am critical of the two main steps of Beecher's proposal, that is the choice of Hockett's design features of language as a starting point, and the presentation of the argument related to the conflict of interests.

## Cognitive Prerequisites for Language Are More Important Than Design Features

Beecher begins his argument by observing that the communication of birds displays a number of key features adapted from Charles Hockett's (1959) classic set of design features of language. Admittedly, Hockett's system is still the most widely used yardstick of comparing human and non-human communication systems, but after over 60 years it has become theoretically obsolete, and assuming it as a point of departure here is unfortunate for several reasons.

Most importantly, the relevance of the system of design features of language to the main thesis of the paper is only indirect. Beecher's main proposal is that what prevents non-human animals from developing a simple language is a lack of extreme social interdependence, even though many species may have the requisite cognitive abilities. Although I agree with this position, it entails that what truly matters is cognitive abilities rather than design features, which in turn makes Beecher's carefully argued interim conclusion—that many animal communication systems have many of the design features of language—orthogonal to his main argument. This is particularly so that Hockett's system concerns the structural and functional properties of the communicative code and is entirely non-cognitive (which, incidentally, is a strong reason to question its applicability to language evolution research, see in particular Wacewicz and Zywiczyński, 2015). In short, the construction of Beecher's argument calls for addressing cognition directly; instead it is only done via the roundabout route of design features, and the relevant cognitive capacities have not been discussed nor identified. This point is far from trivial, since several cognitive capacities considered as evolutionary preconditions for language have been argued to be uniquely human, such as advanced executive functions (e.g., Adornetti, 2016) or advanced intersubjectivity and triadic bodily mimesis (e.g., Zlatev, 2014).

It should also be noted that Hockett's system misses important features of language that make for the truly crucial differences from the communication of other animals, and while making up for these shortcomings is possible, it often results in terminological problems. As one example, a critically important feature of language is its open-ended semantics (cf. e.g., Arbib, 2012), which depends on the domain-generality of human communication—it is semantically universal in the sense of covering any thematic domain, in contrast to narrowly defined domains for many animals systems, such as food calls or alarm calls. Although Beecher does consider this property, he discusses it under “productivity,” which on his account unfortunately conflates three distinct properties of communicative systems: semantic universality, duality of patterning, and productivity in its prototypical meaning of the generative potential of language for structural novelty.

## Conflict of Interests and a Platform of Trust

As a second, and central, condition for developing language, Beecher identifies “near-absent” or even “zero” conflict of interest between communicators. Beecher's focus on game-theoretic explanations, with conflict of interest as a key explanatory variable, is certainly valuable and productive; however—as pointed out in another commentary (Penn and Számadó, 2021)—the requirement that communicators have only minimal or zero conflict of interest is both too strong and unrealistic. Contra Beecher, the challenge for explaining language evolution is not how people have got to have near-absent conflict of interests, because they clearly have not: situations involving a different ordering of preferences between human agents are as ubiquitous now as they undoubtedly must have been in our evolutionary past. Rather, the challenge seems to lie in explaining how humans managed to evolve language *in spite of* non-zero conflict of interests, that is, under conditions that signaling theory predicts language-like systems of large-scale, cheap but honest information donation are not evolvable.

A promising direction is to openly admit this dissociation between general behavior and communicative behavior: while humans clearly do not have completely aligned interests, communicatively they behave *as if* they did. A proposal that captures this is a Platform of Trust, which is defined as “a social niche in which large-scale cheap but honest communication is possible because messages tend to be trusted as a default” (Wacewicz and Zywiczyński, 2018, p. 172), but in terms of the explanatory principle of alignment of interest it can be reformulated as “*as-if* alignment of interests between human communicators.” Importantly, “Platform of Trust” is neutral on how this communicative alignment of interests arose in human evolution. In other words, it is not an explanatory proposal but an explanatory target, in that it is not itself a scenario of language emergence but rather a necessary constraint for any such scenario. However, having well-defined explanatory targets is conducive to better scenarios, which—crucially—take seriously both the uniqueness of human language and the constraints that signaling theory imposes on *all* systems of communication. This is in line with Beecher's main point, which instead of the already almost universally appreciated cognitive preconditions for language prioritizes looking into the underappreciated factors relevant to the often divergent interests of the communicators. Explanations of the stability of honest communication in human societies in the face of a partial conflict of interests between the communicating humans are likely to refer to mechanisms such as epistemic vigilance (Sperber et al., 2010), gossip (Dunbar, 1996), or reputation formation through indirect reciprocity (Nowak and Sigmund, 2005).

## Author Contributions

SW wrote the paper.

## Funding

This research was supported by the Polish National Science Center under grant agreement UMO-2019/34/E/HS2/00248.

## Conflict of Interest

The author declares that the research was conducted in the absence of any commercial or financial relationships that could be construed as a potential conflict of interest.

## Publisher's Note

All claims expressed in this article are solely those of the authors and do not necessarily represent those of their affiliated organizations, or those of the publisher, the editors and the reviewers. Any product that may be evaluated in this article, or claim that may be made by its manufacturer, is not guaranteed or endorsed by the publisher.
